# Use of FDG-PET in Radiation Treatment Planning for Thoracic Cancers

**DOI:** 10.1155/2012/609545

**Published:** 2012-05-14

**Authors:** Katsuyuki Shirai, Akiko Nakagawa, Takanori Abe, Masahiro Kawahara, Jun-ichi Saitoh, Tatsuya Ohno, Takashi Nakano

**Affiliations:** ^1^Gunma University Heavy Ion Medical Center, Maebashi, Gunma 371-8511, Japan; ^2^Department of Radiation Oncology, Gunma University Graduate School of Medicine, Maebashi, Gunma 371-8511, Japan

## Abstract

Radiotherapy plays an important role in the treatment for thoracic cancers. Accurate diagnosis is essential to correctly perform curative radiotherapy. Tumor delineation is also important to prevent geographic misses in radiotherapy planning. Currently, planning is based on computed tomography (CT) imaging when radiation oncologists manually contour the tumor, and this practice often induces interobserver variability. F-18 fluorodeoxyglucose positron emission tomography (FDG-PET) has been reported to enable accurate staging and detect tumor extension in several thoracic cancers, such as lung cancer and esophageal cancer. FDG-PET imaging has many potential advantages in radiotherapy planning for these cancers, because it can add biological information to conventional anatomical images and decrease the inter-observer variability. FDG-PET improves radiotherapy volume and enables dose escalation without causing severe side effects, especially in lung cancer patients. The main advantage of FDG-PET for esophageal cancer patients is the detection of unrecognized lymph node or distal metastases. However, automatic delineation by FDG-PET is still controversial in these tumors, despite the initial expectations. We will review the role of FDG-PET in radiotherapy for thoracic cancers, including lung cancer and esophageal cancer.

## 1. Introduction

Radiotherapy plays an important role in the treatment of thoracic cancers, such as non-small-cell lung cancer (NSCLC), small-cell lung cancer (SCLC), and esophageal cancer [1, 2]. Recent advances in accurate diagnosis improve the practice of curative radiotherapy, because patients with unsuspected metastases may avoid unnecessary local therapies and receive necessary systemic treatment. Accurate delineation of tumor volume is also important to prevent geographic misses in treatment planning. Indeed, an underestimation of tumor extension will result in tumor recurrence. In contrast, overestimation of the extension may increase unnecessary side effects. Therefore, delineation of tumor volumes is a crucial factor in curative radiotherapy.

 Currently, treatment planning is based on computed tomography (CT) imaging to contour the tumor. Tumor delineation is manually performed by each radiation oncologist in clinical practice, which leads to interobserver variability in tumor delineation. Accurate delineation of tumor volume requires the identification of anatomic borders of tumors based on accurate diagnosis. F-18 fluorodeoxyglucose positron emission tomography (FDG-PET) and PET/CT have been reported to enable accurate staging and to detect the tumor extension compared with CT alone [3]. FDG-PET imaging has many potential advantages in radiotherapy planning, because it can add biological information combined with anatomical information by CT imaging. Radiotherapy planning based on FDG-PET is expected to decrease the interobserver variability amongst thoracic radiation oncologists. In addition to accurate staging, FDG-PET has the potential to improve radiotherapy planning by its precise delineation of primary tumor and lymph nodes. Indeed, FDG-PET has been investigated as tracer for radiotherapy planning in many cancer types [4]. In this paper, we focus on the role of FDG-PET in radiotherapy for thoracic cancers, including NSCLC, SCLC, esophageal cancer, and breast cancer.

## 2. Non-Small-Cell Lung Cancer (NSCLC)

### 2.1. Staging

 Thoracic radiotherapy is a key modality of the management for NSCLC patients. Accurate staging is crucial for lung cancer patients because treatment strategy and prognosis drastically differ according to clinical stage. CT imaging contributes to the initial determination for the staging of NSCLC and provides morphologic information of the disease extension. Currently, FDG-PET is performed to diagnose the stage and evaluate the effects of the treatment in clinical use for NSCLC patients. One of the important roles of FDG-PET is to detect unsuspected lymph nodes or extrathoracic metastases [5, 6]. A prospective study has reported that FDG-PET detected unsuspected metastasis in 20% of candidates for radical radiotherapy, changing their treatment strategies [7]. Furthermore, the same authors showed that early mortality rate was low in patients staged by FDG-PET compared with those staged by conventional imaging, on the basis of their accurate staging [8]. Another prospective study demonstrated that curative radiotherapy in 27% of patients was not qualified by FDG-PET because of distant metastasis or extensive locoregional disease [9]. In this study, 32% of patients were upstaged after PET staging. Bradley et al. also reported that FDG-PET altered radiation planning in over 50% of patients staged by CT imaging, due to the detection of unsuspected nodal disease, metastatic disease, or tumor extension from atelectasis [10]. As seen in these findings, the incorporation of FDG-PET improves the accuracy of staging and patient selection, providing better treatment strategy including radiotherapy planning.

### 2.2. FDG-PET for Tumor Volume Delineation

 FDG-PET is increasingly performed to define tumor volume for radiotherapy in patients with NSCLC. Accurate contouring of organs is important for radiotherapy and PET imaging is currently expected to be useful in the delineation of gross tumor volume (GTV) [11]. In this section, we reviewed the published literature on delineation of GTV in NSCLC patients.

 Several methods have been examined for GTV delineation after the introduction of FDG-PET (Table 1) [12–25]. Visual interpretation is a simple method to incorporate PET information with CT imaging. However, this method arbitrarily depends on the window levels set of the PET images. Therefore, the display of PET data should be standardized.

Several articles have reported the use of standardized uptake volume (SUV) in GTV identification. Paulino and Johnstone have suggested using SUV of 2.5 to autocontour GTV [12]. Hong et al. compared target volume delineation by FDG-PET with that by CT imaging in 19 patients with NSCLC, concluding that SUV of 2.5 should be used for radiotherapy planning in NSCLC patients [18]. Another method was the use of the ratio of SUV. Erdi et al. analyzed a phantom with lung lesion by source-to-background ratios [13]. They applied this data to 10 patients with 17 primary or metastatic lung lesions, concluding that the source-to-background ratio was useful in the definition of tumor volumes. Nestle et al. compared GTVs obtained from four different methods: visually (GTVvis), applying a 40%-threshold of SUV max (GTV40), using an autocontour of SUV 2.5 (GTV2.5), and using an algorithm by phantom measurements (GTVbg) in 25 patients with NSCLC [14]. In the results, GTVvis, GTV2.5, and GTVbg were relevant to the CT-derived GTV, whereas GTV40 appeared unsuitable for target volume delineation.

 Recently, some studies reported a new gradient-based method relied on the water shed transform and hierarchical cluster analysis. This method provided denoised and deblurred images with an edge-preserving filter and a constrained iterative deconvolution algorithm, leading a better estimation of the gradient intensity. Wanet et al. compared a gradient-based segmentation method, a source-to-background ratio method, and a 40% to 50% of SUV max method with surgical specimens of 10 patients with NSCLC [15]. All patients underwent image inspections before surgery. Among several methods, the gradient-based method achieved the best results, with the lowest average error and smallest standard deviation. Werner-Wasik et al. conducted the same experiment using the digital thorax phantom and evaluated the accuracy and consistency of three methods: gradient-based PET segmentation, manual, and constant threshold methods [16]. They concluded that the gradient-based method was the most accurate with the least systematic bias at any phantom size.

 Despite these several techniques for the delineation of tumor volume having been reported in recent years, a gold standard method has not been established to contour GTV automatically. However, FDG-PET has the additional information that aids radiation oncologists to delineate GTV, indicating that FDG-PET should be utilized when tumor volume is delineated in NSCLC patients.

### 2.3. Reduction of Interobserver Variability

Interobserver variability in regard to GTV definition is a serious problem in the treatment planning for NSCLC [26, 27]. Caldwell et al. prospectively evaluated the role of FDG-PET in interobserver variability in 30 NSCLC patients [28]. Three radiation oncologists contoured GTV using CT alone or FDG-PET registered CT (PET/CT). The mean coefficient of variation for GTV based on PET/CT was significantly smaller than CT alone (*P* < 0.01). Recently, van Barrdwijk et al. also reported that PET/CT-based autocontouring decreased interobserver variability in delineation of the primary tumor and nodal legions [29]. These studies indicated that FDG-PET can reduce interobserver variability in radiotherapy treatment planning.

### 2.4. Role of FDG-PET in Stereotactic Radiotherapy for Early-Stage NSCLC

 Currently, more patients have been treated by stereotactic body radiotherapy (SBRT) for early-stage NSCLC [1, 2]. Recent studies reported the role of FDG-PET in SBRT for NSLCL, such as staging, treatment response, and planning. Henderson et al. performed serial planned FDG-PET (before and after SBRT) in stage I NSCLC patients treated by 60–66 Gy in three fractions [30]. In their study, substantial proportion of patients had moderately elevated SUVmax at 52 weeks after SBRT without evidence of local failure, suggesting that slight elevation of SUVmax should not be surrogate for local failure. Andratschke et al. performed SBRT that consisted of 3–5 fractions with 7–15 Gy per fraction in 92 stage I NSCLC patients [31]. Most patients were staged according to FDG-PET. Isolated regional recurrence was observed in only 7.6%. The authors concluded that elective irradiation can safely be omitted in stage I NSCLC after accurate nodal staging with PET-CT.

 Takeda et al. evaluated the correlation between pretreatment SUVmax on FDG-PET and local recurrence in 95 patients with NSCLC [32]. Total dose of SBRT was 40–50 Gy in five fractions. Two-year local recurrence rates for lower SUVmax (<6.0) and higher SUVmax (≥6.0) were 93% and 42%, respectively (*P* < 0.001). Multivariate analysis showed that only the SUVmax of a primary tumor was a significant predictor for local recurrence (*P* = 0.002). The authors concluded that a high SUVmax might be considered for dose escalation to improve local control in NSCLC. Hoopes et al. also reported that 58 patients with medically inoperable stage I NSCLC treated by SBRT [33]. Total doses ranged from 24 to 72 Gy in three fractions. In their study, pretreatment SUVmax did not predict three-year overall survival or local control. Coon et al. performed SBRT with a dose of 60 Gy in three fractions for stage I NSCLC (*n* = 26), recurrent lung cancer (*n* = 12), and solitary lung metastases (*n* = 13) [34]. Most patients received PET-CT before and after SBRT. PET-CT was valuable in staging, treatment planning, but no correlations were found between pretreatment SUVmax and treatment response, disease progression-free survival, and overall survival.

 These findings suggest that FDG-PET is useful for accurate staging and treatment planning in stage I NSCLC treated by SBRT. However, it has been controversial whether pretreatment SUVmax is prognostic factor for treatment outcomes, such as local control and overall survival. Prospective studies are warranted to establish the role of FDG-PET in SBRT for NSCLC patients.

### 2.5. Role of FDG-PET on Evaluation for Tumor Recurrence

For patients with NSCLC, local failure after radiation therapy is a significant issue. Sura et al. reported FDG-PET as a method to assume the pattern of local failure after radiation therapy [35]. They analyzed the data of 26 patients with 34 recurrent legions that were contoured using a fixed threshold of 42% of the maximum SUV by post-RT PET/CT. The result showed that the pattern of recurrence depended on the radiation dose. At a total dose of <60 Gy, most recurrences were within target volume. At a total dose of ≧60 Gy, recurrences were within the marginal zone of the target volume.

 Similarly, Abramyuk et al. also reported the detection of recurrence using PET/CT [36]. They evaluated whether PET/CT is capable of predicting the location of recurrence in patients with NSCLC, especially for determining high-risk area in tumor. Ten patients with local failure of NSCLC were analyzed. After radiation therapy, 2 patients' lesions had a complete metabolic response and 8 patients' lesions showed reduced SUV. However, all patients were diagnosed with recurrence 12 months after the radiation therapy. The location of recurrence was mostly in the most active metabolic lesion (threshold > 35% of SUV max) of primary tumor. This result may be applied to further radiation therapy of additional doses to the area of higher FDG uptake.

### 2.6. Role of FDG-PET in Elective Nodal Irradiation

 Elective nodal irradiation to the mediastinal lymph regions in the treatment of stage III NSCLC patients is still a controversy [37, 38]. Rosenzweig et al. retrospectively evaluated the failure rates in uninvolved nodal regions with involved-field radiotherapy for inoperable 524 patients with NSCLC. The 2-year elective nodal control rate was 91% and the authors concluded that involved-field radiotherapy did not cause a significant amount of failure in lymph node regions [39]. RTOG0117 also performed involved-field radiotherapy to perform high-dose irradiation without severe side effects [40]. However, some clinicians have raised the concern that omission of elective nodal irradiation requires a further discussion, because recurrence of lymph nodes is usually fatal and microscopic metastasis of lymph nodes occurs substantially in advanced NSCLC [38].

FDG-PET can contribute to accurate evaluation of nodal lesions, compared with CT alone [41, 42]. De Ruysscher et al. conducted a prospective study of involved-field radiotherapy based on FDG-PET for 44 patients [43]. In this study, only 1 patient (2.3%) developed an isolated nodal failure, and the authors concluded that FDG-PET was useful in the involved-field radiotherapy for NSCLC patients. Furthermore, Kolodziejczyk et al. reported that FDG-PET should be used even in the elective nodal irradiation planning for NSCLC, because elective nodal irradiation may not compensate the unsuspected mediastinal lymph nodes [9]. It is controversial whether elective nodal irradiation can be safely omitted by FDG-PET or not. However, this strategy is extremely attractive because it allows radiation-dose escalation without severe side effects. Further studies are warranted to establish the utility of FDG-PET in NSCLC for radiation planning.

## 3. Small-Cell Lung Cancer (SCLC)

### 3.1. Staging

SCLC accounts for 20–25% of all newly diagnosed lung cancers. SCLC often represents an aggressive clinical course and high incidence of distant metastasis because of rapid tumor growth. Despite aggressive treatment, the prognosis remains poor [44].

 Although accurate staging is essential for determining the treatment strategy in SCLC, it is difficult to evaluate the extension of disease accurately, and especially mediastinum lymph node metastases. Fischer et al. prospectively examined the role of PET/CT compared with standard staging (CT and bone scintigraphy) in 29 SCLC patients [45]. In their study, PET/CT changed the stage in 5 of the 29 patients (17%), with the authors concluding that PET/CT improves the accuracy of staging in SCLC. In other studies, 8.3% to 9.5% of limited-disease SCLC staged by the conventional staging procedures was upstaged to extended-disease SCLC after FDG-PET information was incorporated [46, 47]. Arslan et al. evaluated the accuracy and overall survival staged by FDG-PET or CT imaging [48]. PET scan upstaged 9 (36%) in 25 patients and downstaged 2 (8%) in 25 patients who were staged by CT imaging. Furthermore, FDG-PET staging predicted significant survival difference (*P* = 0.019), while CT imaging did not (*P* = 0.055). These studies recommended that FDG-PET should be performed for initial staging in limited-disease SCLC patients.

### 3.2. FDG-PET for Tumor Volume Delineation

 Compared with NSCLC, few studies have been performed regarding the delineation based on FDG-PET for SCLC. FDG-PET in SCLC patients may improve the delineation of tumor volume as well as NSCLC.

### 3.3. Involved Field-Based FDG-PET Planning

For limited-disease SCLC, elective nodal irradiation for mediastinal lymph node regions has been considered necessary to reduce lymph node failure. However, some clinicians have attempted to avoid elective nodal irradiation because extended radiotherapy volume leads to severe side effects. Baas et al. conducted a phase II study of involved-field radiotherapy for limited-disease SLCL staged by CT imaging [49]. They reported median survival of 19.5 months and low incidence of adverse effects. De Ruysscher et al. also conducted a phase II trial of involved-field irradiation based on CT imaging for limited-disease SCLC [50]. They evaluated overall survival and isolated nodal failure defined as recurrence in regional nodes outside the target volume in the absence of in-field failure. In their study, isolated mediastinal lymph node recurrence was unexpectedly high (11%). Given these findings, a report from the International Atomic Energy Agency (IAEA) consultants' meeting indicated that involved-field irradiation in SCLC was controversial and should be performed in a prospective clinical trial [51].

 Currently, FDG-PET is expected to determine whether elective nodal irradiation is necessary or not. Two recent studies have shown the usefulness of FDG-PET in omitting elective nodal irradiation in SCLC [52, 53]. van Loon et al. conducted a prospective study of involved-field irradiation on the basis of FDG-PET for 60 patients with limited-disease SCLC [52]. Median actuarial overall survival was 19 months and isolated nodal failures was low (3%). The authors concluded that treatment planning based on FDG-PET could decrease isolated nodal failures, compared with their previous results (isolated nodal failure: 11%) treated by CT-based involved-field irradiation. Shirvani et al. reported that involved-field irradiation by intensity-modulated radiotherapy (IMRT) for 60 patients with limited-disease SLCL was staged by FDG-PET [53]. In this study, the 2-year actuarial overall survival was 58% and isolated elective node was observed in only one patient (3%). These studies concluded that elective nodal irradiation could be safely omitted by FDG-PET staging in SCLC. Avoidance of mediastinal lymph nodes that are PET-negative can lead to (1) a reduction of toxicity with the same radiation dose or (2) dose escalation with the same toxicity. Although involved-field irradiation based on FDG-PET is an attractive treatment in SCLC, there is not enough information to recommend the strategy. Further prospective studies are required to determine whether elective nodal irradiation can be safely omitted by FDG-PET staging in SCLC.

 Another possible role of FDG-PET in SCLC is in the evaluation of the therapeutic response. A preliminary study showed that FDG-PET could predict the outcome of treatment by radiotherapy or chemotherapy [54].

 In conclusion, the role of FDG-PET in SCLC has been established for staging use. Although the use of FDG-PET in radiation treatment planning for SCLC is still controversial, involved-field irradiation based on FDG-PET is an attractive strategy. FDG-PET-based treatment planning will change the strategy for limited-disease SCLC.

## 4. Esophageal Cancer

In the treatment of esophageal cancer, radiotherapy is commonly used in combination with chemotherapy. Currently, radiotherapy requires accurate target volume definition based on treatment planning by CT scan. Although planning-CT-based target volume definition is considered the gold standard in esophageal cancer, applying FDG-PET to treatment planning may have several advantages, such as accurate staging and delineation of tumor.

### 4.1. Staging

 FDG-PET has been considered useful in the staging process of esophageal cancer [55]. Flamen et al. reported that 70 primary tumors of 74 patients were detected by FDG-PET, with a sensitivity of 95% [56]. However, 4 patients with T1 lesions were not detected by FDG-PET. The authors showed that FDG-PET had a higher accuracy for stage IV disease compared with conventional modalities (82% versus 64%, *P* = 0.004). Van Vliet et al. performed a meta-analysis to evaluate the diagnostic performance of CT and FDG-PET in staging of esophageal cancer [57]. The sensitivities of CT and FDG-PET for regional lymph node metastases were 0.50 and 0.57, respectively, and their specificities were 0.83 and 0.85, respectively. The detection of distant metastases by FDG-PET was significantly higher than CT. The authors concluded that each modality plays a distinctive role in the detection of esophageal cancer.

### 4.2. FDG-PET for Tumor Volume Delineation

Accurate delineation of esophageal tumor is important for successful radiotherapy. The additional information provided by FDG-PET is expected to improve tumor delineation and accurate staging of lymph nodes and distant metastases. Several studies have investigated the optimal method for delineating the target volume by FDG-PET. Most studies used visualized interpretation for tumor delineation [58–61]. Moureau-Zabotto et al. evaluated the effect of the addition of FDG-PET to CT in tumor delineation of 34 esophageal cancer patients [59]. FDG-PET decreased the length of GTV in 12 patients (35%) and increased the length in 12 patients (35%).

 Konski et al. used SUV 2.5 to delineate the tumor extension and evaluated the CT-based tumor length in 25 esophageal cancer patients [62]. The authors concluded that FDG-PET provides additional information for the identification of GTV. Zhong et al. compared FDG-PET-based tumor length with surgical specimens and showed that SUV 2.5 seemed closest to the pathological length [63]. Hong et al. reported that automated interpretation of FDG-PET using mean activity of the liver plus 2 standard deviations likely affect target definition [64]. Recently Vali et al. compared 11 different methods: SUV 2.0, 2.5, 3.0, and 3.5; SUV Max 40%, 45%, and 50%; mean liver SUV plus 1, 2, 3, and 4 standard deviations [65]. The authors concluded that the use of a threshold of approximately 2.5 was the optimal method for the delineation of GTV in esophageal cancer, regardless of SUV thresholding method.

 Recent studies indicated that SUV 2.5 may be an optimal threshold, but autocontouring using this threshold is not satisfactory. Further investigations are required to utilize FDG-PET in the tumor delineation for esophageal cancer in clinical use.

### 4.3. Interobserver Variability

Another method to utilize FDG-PET in treatment planning is to reduce interobserver variability. Schreurs et al. evaluated the tumor volumes delineated by FDG-PET in 28 esophageal cancer patients by three radiation oncologists [66]. The authors concluded that FDG-PET might improve target volume definition with less geographic misses, but the effects on interobserver variability were not significant. Vesprini et al. compared FDG-PET/CT with CT alone for the identification of GTV in 10 patients with esophageal cancer by 6 radiation oncologists [60]. The addition of FDG-PET significantly decreased both inter- and intraobserver variability.

### 4.4. The Effect of FDG-PET on Radiotherapy Planning

 Muijs et al. reported that the additional use of FDG-PET led to the modification of CT-based radiotherapy planning in 57% of esophageal cancer patients [61]. Furthermore, they showed that FDG-PET significantly changed the radiation dose for organs at risk, such as heart and lung. The additional information provided by FDG-PET has the possibility of improving the local control due to less geographic misses. However, there are no studies that showed whether FDG-PET affects survival or local control rate. Further studies are warranted to establish the role of FDG-PET in highly accurate radiotherapy planning for esophageal cancer.

## 5. Prospects of PET Imaging for Thoracic Cancers

### 5.1. Respiratory-Gated PET Imaging

One of the limitations in the use of PET imaging in radiotherapy planning is the low spatial resolution. Generally, the spatial resolution of current PET scanners (6–8 mm) is inferior to that of modern CT scanners (1 mm) [67]. Furthermore, because PET requires several minutes to perform the imaging, tumor motion due to respiration or cardiac action deteriorates PET images and increases diagnostic errors in thoracic cancers. In fact, it is one of the major limitations of FDG-PET in several tumor types and has been largely responsible for the slow acceptance of FDG-PET when major treatment decisions are made on the basis of a negative FDG-PET. Respiratory-gated PET imaging is currently expected to improve the quantification of PET [68]. Sakaguchi et al. reported that gated imaging acquisition improved the quality of FDG-PET imaging in a moving phantom model [69]. Daouk et al. evaluated 48 pulmonary nodules in 43 patients by respiratory-gated PET and ungated method [70]. Gated imaging had higher sensitivity and specificity than the ungated method, especially for smaller lesions located in lower lobes. These findings suggested that respiratory-gated PET should be performed for patients with thoracic cancers with respiratory motions.

### 5.2. FLT-PET

Other nuclides have been investigated to overcome the problems of FDG-PET. Currently, 3′-deoxy-3′-(18)F-fluorothymidine (FLT), a PET imaging marker of proliferation, has been introduced as an alternative to FDG for lung cancer [71]. Yang et al. compared the diagnostic efficacy of FLT-PET and FDG-PET in NSCLC [72]. The sensitivities of FLT- and FDG-PET for primary tumor were 74% and 94%, respectively. In contrast, FLT-PET showed better specificity and accuracy than FDG-PET for lymph nodes. Although the use of FLT-PET is still at an investigational level, further studies are expected to continue to evaluate its efficacy.

 When PET is used for radiotherapy planning, a range of uncertainties related to technical, physical, biological, and analytical factors must be considered. Further investigations are warranted to establish highly accurate radiotherapy based on PET imaging for thoracic cancers.

## 6. Summary

FDG-PET plays a pivotal role in accurate staging and selection of patients to be treated by radiotherapy. Furthermore, FDG-PET improves radiotherapy volume and enables dose escalation without severe side effects in lung cancer patients. The main advantage of FDG-PET for esophageal cancer patients is the detection of unrecognized lymph nodes or distal metastases. While FDG-PET was initially expected to result in more accurate target delineation, its efficacy remains controversial and delineation by FDG-PET should not be practiced in clinical use at this stage. Further studies are required to confirm the advantage of FDG-PET in radiotherapy planning for thoracic cancers.

## Figures and Tables

**Table 1:** Target volume delineation in NSCLC.

Author	Year	Patients	Methods of delineation	Conclusion
Nestle et al. [14]	2005	25	Visual 40% of SUV max ≧ 2.5 SUV Phantom algorithm	Visual, SUV of 2.5, and phantom algorithm were associated with GTV delineated by CT.
Biehl et al. [17]	2006	20	20%–40% of SUV max	No single threshold delineating PET provides accurate volume definition, compared with that provided by CT.
Hong et al. [18]	2007	19	SUV ≧ 2.5 40% of SUV max	This study recommended using SUV ≧ 2.5 for radiotherapy planning in non-small-cell lung cancer.
van Baardwijk [19]	2007	23	Source-to-background ratio	Source-to-background ratio-based autodelineation was strongly correlated microscopic diameter of primary tumor (correlation coefficient = 0.90).
Visser et al. [20]	2008	13	50% of SUV max 50% of glucose metabolic rate	Tumor volumes from glucose metabolic rate were significantly smaller than SUV-based volumes.
Rodríguez et al. [21]	2010	40	40% of SUV max	Lymph nodes could be delineated in accordance with tumor uptake when lymph nodes/tumor SUV max ratio was ≤25%.
Devic et al. [22]	2010	31	Visual 15%, 40% of SUV max	Not dependent on the thresholding method used.
Vinod et al. [23]	2010	5	Visual	Effects of FDG-PET on normal tissue complication and tumor control cannot be predicted.
Wanet et al. [15]	2011	10	Gradient-based method Source-to-background ratio 40%, 50% of SUV max	Gradient-based method best estimated true tumor volume.
Warner-Wasik et al. [16]	2011	Phantom	Gradient-based method	Gradient-based method was most accurate and consistent technique for target volume contouring.
			Visual ≧2.5 SUV	
			Source-to-background ratio	
Fleckenstein et al. [24]	2011	32	Source-to-background ratio	FDG-PET confined target volume definition was associated with low risk of isolated nodal recurrences.
Lin et al. [25]	2011	37	Visual	There was correlation between GTV based on FDG-PET and excised surgical specimen.
